# Starch-Based Aerogels Obtained via Solvent-Induced Gelation

**DOI:** 10.3390/gels6030032

**Published:** 2020-09-19

**Authors:** Mirelle Dogenski, Pavel Gurikov, Victor Baudron, J. Vladimir de Oliveira, Irina Smirnova, Sandra R. S. Ferreira

**Affiliations:** 1 Department of Chemical and Food Engineering, Federal University of Santa Catarina, Florianópolis 88040-900, Brazil; jose.vladimir@ufsc.br; 2 Laboratory for Development and Modelling of Novel Nanoporous Materials, Hamburg University of Technology, Eißendorfer Straße 38, 21073 Hamburg, Germany; 3 Institute of Thermal Separation Processes, Hamburg University of Technology, Eißendorfer Straße 38, 21073 Hamburg, Germany; victor.baudron@tuhh.de (V.B.); irina.smirnova@tuhh.de (I.S.)

**Keywords:** starch-based aerogels, dimethyl sulfoxide, solvent-induced gelation, solubility parameters

## Abstract

In this work, the ability of several solvents to induce gel formation from amylomaize starch solubilized in dimethyl sulfoxide (DMSO) was investigated. The formed gels were subjected to solvent exchange using ethanol and dried with supercritical carbon dioxide (sc-CO_2_) to obtain the aerogels. The influence of starch concentration (3–15 wt%) and solvent content (20–80 wt%) on gel formation was also studied. It was demonstrated that the gelation of starch in binary mixtures of solvents can be rationalized by Hansen Solubility Parameters (HSP) revealing a crucial hole of hydrogen bonding for the gel’s strength, which is in agreement with rheological measurements. Only the addition of water or propylene glycol to starch/DMSO solutions resulted in strong gels at a minimum starch and solvent content of 7.5 wt% and 50 wt%, respectively. The resulting aerogels showed comparably high specific surface areas (78–144 m^2^ g^−1^) and low envelope densities (0.097–0.203 g cm^−3^). The results of this work indicate that the HSP parameters could be used as a tool to guide the rational selection of water-free gelation in starch/DMSO systems. In addition, it opens up an attractive opportunity to perform starch gelation in those solvents that are miscible with sc-CO_2_, avoiding the time-consuming step of solvent exchange.

## 1. Introduction

Starch is one of the most abundant biopolymers and the major carbohydrate reserve in higher plants. In vegetal tissues, starch it is stored as granules, and the major compounds (98–99 wt%) are amylose and amylopectin. Amylose is a linear polymer composed of α-D-glucopyranose linked α-(1,4), while amylopectin is highly branched at the positions α-(1,6) besides the linkages in α-(1,4). Although starch can be found in almost all vegetables, the main commercial sources are corn, wheat, potato, cassava, and rice. This important biopolymer is largely applied as thickening and gelling agent in food and non-food products [1,2]. Moreover, owing to its biodegradability and biocompatibility, starch has been widely associated with the development of new versatile eco-friendly porous materials, in particular, aerogels [3,4,5,6].

Starch-derived aerogels, low-density mesoporous solids with high specific surface area, are commonly prepared in three steps: hydrogel formation, solvent exchange using ethanol, and supercritical drying with carbon dioxide (CO_2_) [3,7]. Aerogel properties are highly dependent on the source of starch and its concentration, degree of gelatinization, and retrogradation [3,8]. Starch-derived aerogels are suggested for many applications such as carriers for active compounds [8,9,10,11], thermal insulators [12,13,14], tissue engineering [15,16], as templates for making novel materials [17], and adsorbents [18].

Starch gels are predominantly formed in aqueous media under thermal gelation through the so-called gelatinization and retrogradation processes [1]. Depending on the amylose content, gelatinization temperatures up to 120 °C are necessary to achieve a satisfactory disruption of starch granules (e.g., for amylomaize starches) [19]. It is of high interest for the aerogel technology to eliminate aqueous medium at the gelation step and to be able to perform gelation in those solvents that are miscible with supercritical carbon dioxide. This would allow excluding a time-consuming step, namely the solvent exchange, and make a next step toward an integrated processing of aerogels [20]. Interestingly, non-aqueous solvents such as DMSO with an ability to form hydrogen bonds can be applied for starch gelation [21]. Dimethyl sulfoxide (DMSO) is an aprotic highly dipolar solvent largely used for solubilization of starch granules in mild conditions (≈80 °C) [22,23,24,25]. McGrane et al. (2004) [21] demonstrated that phase transitions of starch/DMSO mixtures, from liquids to gels, can be achieved by adding a second solvent to reduce the starch–DMSO interactions forming cross-linking bridges between the polymer chains. They elucidated the hole of hydrogen bonding in amylose gelation through rheological measurements in samples containing 10 wt% starch in DMSO and added different classes of solvents such as polyols, alcohols, and urea, besides water. In this work, we expand the list of studied solvents and elucidate the conditions at which gelation occurs with a focus on the subsequent conversion of starch gels into corresponding aerogels. To the best of our knowledge, the latter aspect has never been studied.

To rationalize the outcome of the gelation experiments, we employ the Hansen Solubility Parameters (HSP). In the field of polymer science, the HSP are a powerful empirical tool largely used to predict polymer solubility. More recently, it has been successfully employed to predict organogels formation [26,27,28]. In the HSP approach, three major types of interaction between molecules are taken into account: dispersive interactions (d), dipole–dipole interactions (p), and hydrogen bonding (h). The sum of all individual parameters gives the total solubility parameter (*δ_t_*), or the so-called total Hildebrand solubility parameter: $\delta_{t}^{2}={\;\delta }_{d}^{2}+\delta_{p}^{2}+\delta_{h}^{2}$ [29]. To predict the solubility, the HSP of the solvent and the polymer are compared. Basically, the smaller the distance between the two points in the three-dimensional (3D) Hansen space, the more likely the polymer is compatible with the solvent (it dissolves or swells). Raynal and Bouteiller (2011) [28] first applied the HSP to correlate solvent parameters to the gelation ability of several low molecular weight gelators (LMWG). They demonstrated that while the molecular dissolution requires a solvent that can efficiently compete with the intermolecular gelator interactions, the gelation relies on the establishment of this interaction. Based on that, two regions (dissolution and gelation spheres) can be identified in the 3D Hansen space. The comparison of individual solubility parameters also revealed which kind of interactions (dispersive, dipole–dipole, and hydrogen bonding) were involved in the dissolution and gelation processes [28]. Here, the aim is to extend this approach to understand the interactions between starch and the binary mixtures of solvents with DMSO.

In this scope, we set the goal to explore phase transitions in DMSO solutions of starch when combined with several solvents. The influence of starch concentration, type of solvent, and solvent-to-DMSO mass ratio on gel formation were discussed in terms of visual appearance, solubility parameters, and rheological measurements. Textural properties of aerogels were characterized through nitrogen adsorption/desorption technique and Scanning Electron Microscopy (SEM).

## 2. Results and Discussion

The ability of several solvents to induce gelation in DMSO solutions of starch was first investigated. Samples containing starch concentrations of 3, 5, 7.5, and 10 wt% were prepared with the following solvent-to-DMSO mass ratios: 20:80, 30:70, 50:50, 70:30, and 80:20 *w*/*w*, using stock solutions of 30–37 wt% starch. For samples containing 15 wt% starch, the solvent-to-DMSO mass ratios used were 20:80, 30:70, and 50:50. This was because the stock solutions with starch contents larger than 40 wt% were too viscous to guarantee good homogeneity of mixing at higher solvent-to-DMSO mass ratios.

### 2.1. Effect of the Processing Conditions on Gel Formation

**Figure 1:**
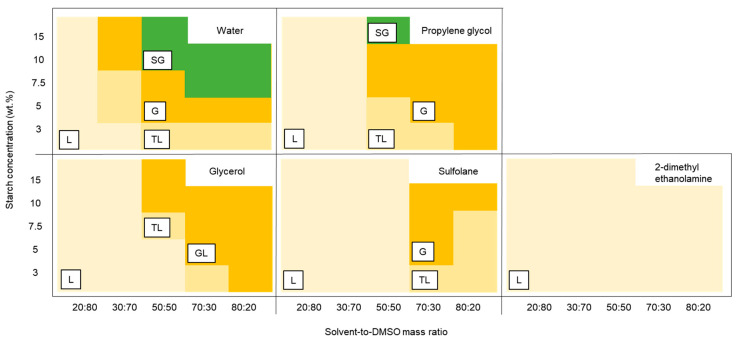
Qualitative phase diagrams of the samples containing different solvents, with increasing starch concentration and solvent-to-DMSO mass ratio. The letters represent the gelation behavior of samples: liquid (L), thick-liquid (TL), gel-like (G), and strong gel (SG).

The effect of the solvent nature, starch concentration, and solvent-to-DMSO mass ratio on gel formation is shown in Figure 1. The qualitative diagrams were constructed according to the classification presented in Section 4.3. The visual appearance and flow behavior of the samples are shown in Table S1, in the Supplementary Materials. In general, the ability to form strong gels increases with rising starch concentration and solvent content. As expected, the type of solvent had a crucial role in the gelation process. Among all the solvents tested in this study, water provided the highest capacity to induce the formation of strong gels: six samples were classified as strong gels from a total of 23 studied (Figure 1). Propylene glycol was also able to induce the formation of a strong gel, but only at 15 wt% starch and a propylene glycol-to-DMSO mass ratio of 50:50. Glycerol and sulfolane yielded samples with gel-like appearance only, which when touched were very sticky (glycerol-added samples) or brittle (sulfolane-added samples). All samples containing 2-dimethyl ethanolamine as solvent showed a liquid-like appearance. From these results, samples were selected for further investigation by rheological measurements and the production of aerogel monoliths.

### 2.2. Gel Formation Rationalized by HSP

**Figure 2:**
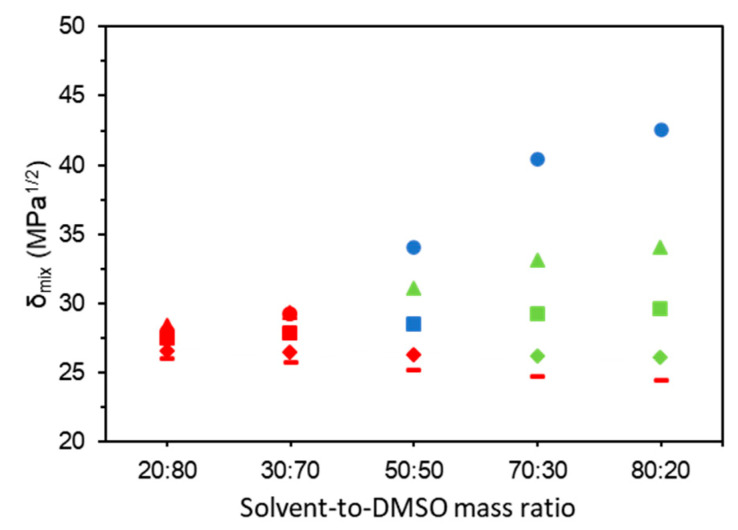
Solubility parameters of the mixture (*δ_m_*) for DMSO with (●) water, (■) propylene glycol, (▲) glycerol, (♦) sulfolane, (-) and 2-dimethyl ethanolamine and gelation behavior of the samples, that is represented by colors: red = liquid; green = gel-like; blue = strong gel.

Figure 2 shows the solubility parameter of the mixture (*δ_m_*) and the gelation behavior obtained for all solvent-to-DMSO mass ratios. The tabular data are presented in Table S2 in the Supplementary Materials. From Figure 2, it is clear that with the increase in the *δ_m_*, there is a tendency to form gel-like and strong gel structures, indicating a reduction in the affinity of starch to DMSO and an increase in the polymer–polymer chain interactions. Following this tendency, it would be expected that glycerol presents a better ability to form strong gels than propylene glycol, but the opposite is observed. As pointed by McGrane et al. (2004) [21], there are some possible reasons for this behavior: (i) the unbonded residual -CH_2_-OH group of glycerol is unlikely to bond to a third amylose molecule due to steric restriction; (ii) all the three alcohol groups form intramolecular bonds with amylose molecules, resulting in an irregular alignment of amylose chains and preventing the gel formation. Reducing the number of hydroxyl groups from three in glycerol to two in propylene glycol seems to favor the polymer–polymer interactions up to a certain point, when starch concentration and propylene glycol-to-DMSO mass ratio were 15 wt% and 50:50 (strong gel appearance), respectively.

The *δ_m_* for binary mixtures of DMSO with sulfolane and 2-dimethyl ethanolamine are between 24.3 and 26.6 MPa^1/2^ (Table 1). To the best of our knowledge, there is only one rough estimation of the total solubility parameters for native starch, which is 24 MPa^1/2^ [30]. No estimations were found for the individual solubility parameters ($\delta_{d}^{2}$, $\delta_{p}^{2}$, and $\delta_{h}^{2}$). Assuming a close value for amylomaize starch, it would explain the fact that the biopolymer remains solubilized in all 2-dimethyl ethanolamine-to-DMSO mass ratios, despite the ability of this solvent to form hydrogen bonds. On the other hand, higher proportions of sulfolane (sulfolane-to-DMSO mass ratios of 70:30 and 80:20) provided gel-like samples. It is known that compounds with *δ_d_* close to 18 are generally in solid state at ambient conditions (20–25 °C), which is the case of sulfolane, *δ_d_* = 18.4 MPa^1/2^ (Table 1) [29]. Thus, the visual aspect of the referred samples would be more associated to the solvent physicochemical properties than a gelation process itself. The great ability of water as solvent on the formation of strong gels would be a reflection of its small molecular size and its structure provided by hydrogen bonding, promoting better inter- and intramolecular interactions through hydrogen bonds between polymer chains than the other solvents [21].

**Figure 3:**
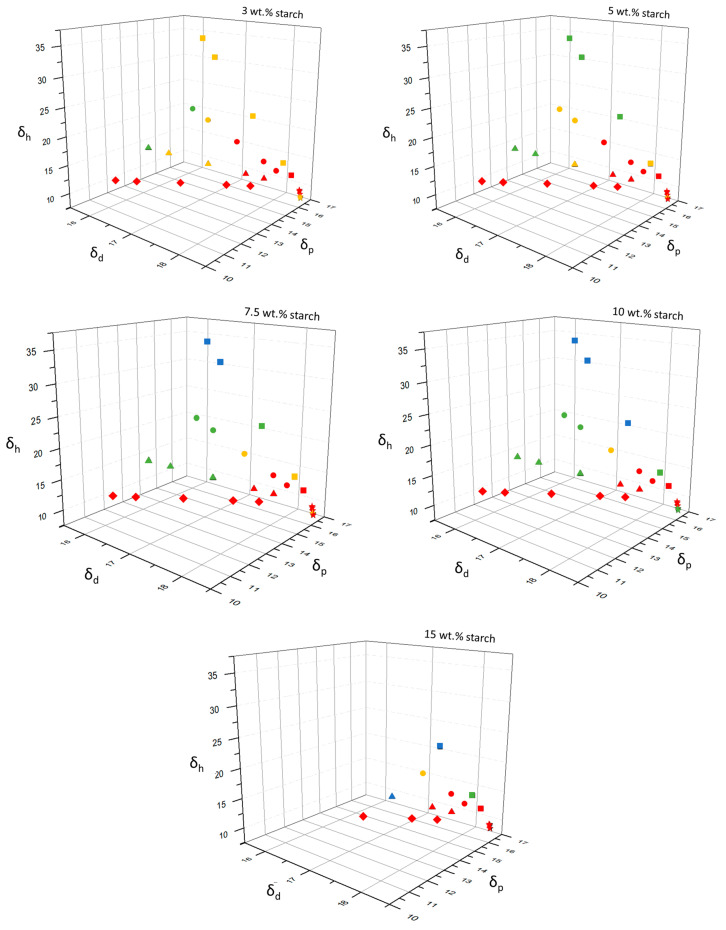
Gelation behavior of starch in various binary mixtures of solvents plotted in 3D Hansen space. The symbols represent the mixtures of DMSO with water (●), propylene glycol (■), glycerol (▲), sulfolane (★), and 2-dimethyl ethanolamine (♦). The colored symbols represent the gelation behavior of starch, in which: red = liquid; yellow = thick liquid; green = gel-like; blue = strong gel.

To better understand the role of each type of chemical interaction on gel formation, the individual solubility parameters (*δ_d_*, *δ_p_*, *δ_h_*) were plotted in the 3D Hansen space for all solvent-to-DMSO mass ratios (Figure 3). It is observed that for all starch concentrations studied, the samples with poor gelation (red-colored symbols in Figure 3) are grouped in a region of both low *δ_h_* and high *δ_d_* and *δ_p_* values, reflecting the role of hydrogen interactions in the formation of gel-like and strong gel structures (green and blue-colored symbols, respectively). We can hypothesize that the most suitable solvents to promote gel formation in a starch/DMSO system should be those with high values of *δ_t_* (>27.6 MPa^1/2^; see Table S2 in the Supplementary Materials) and *δ_h_*. Figure 3 also shows that the increase in starch concentration has a positive influence on gel formation.

### 2.3. Rheological Properties of Gel Formation

**Figure 4:**
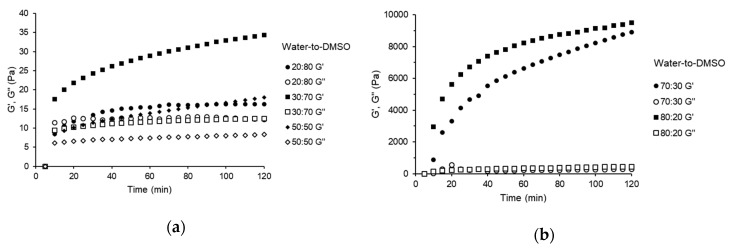
Mechanical spectra of the samples containing 10 wt% starch and different water-to-DMSO mass ratios: (**a**) 20:80, 30:70, and 50:50; (**b**) 70:30 and 80:20.

The mechanical spectra for samples containing 10 wt% starch with various water contents are presented in Figure 4. The figure demonstrates the time dependency of elastic and viscous modulus (G’ and G”, respectively) for water-to-DMSO mass ratios of 20:80, 30:70, 50:50 (Figure 4a), and 70:30, 80:20 (Figure 4b). All samples exhibited a typical gel-like mechanical spectra, with the elastic modulus (G’) dominating over the viscous modulus (G’’) during the observation time (120 min). A similar behavior was detected for samples containing 5, 7.5, and 15 wt% starch (Figures S1–S3, respectively, see Supplementary Materials). The results suggest that the gel strength increases with both starch concentration and water content, which is characterized by the increase in G’ values and the distance between G’ and G”. These results corroborate with the visual assessment of samples shown in Figure 1. Amylose solubilized in DMSO is reported to adopt a helical conformation [23], and with the increase in water content, the polymer conformation gradually changes from tight helical to loose helical, and then to disordered coil. These conformational transitions expose the hydroxyl groups and enable inter and intramolecular interactions between polymeric chains through hydrogen bonds [22]. As a result, the gel strength increases with water content and starch concentration.

**Figure 5:**
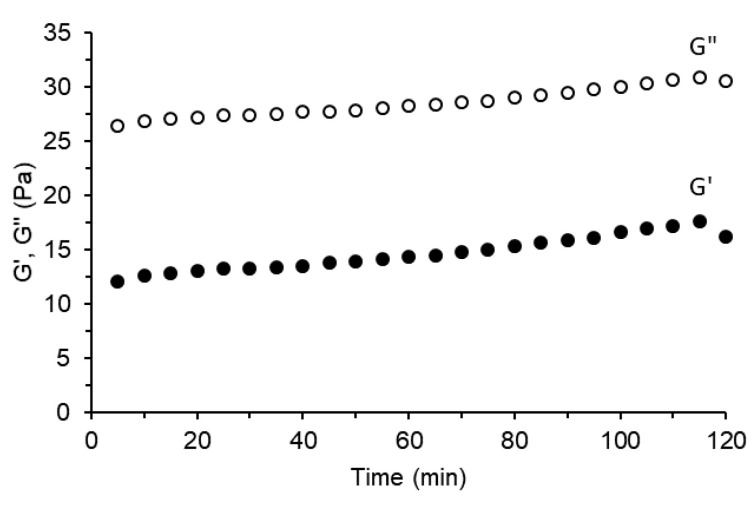
Mechanical spectra of the sample containing 15 wt% starch and propylene glycol-to-DMSO mass ratio of 50:50.

The mechanical spectra for the sample with 15 wt% starch in propylene glycol/DMSO mixture (mass ratio of 50:50) is presented in Figure 5. In this figure, G’ is lower than G”, indicating that a gel-like structure was not formed during the analysis period (120 min). Despite that, propylene glycol provided gel monoliths with good firmness after the hardening period of 24 h. The slower gelling process when compared to water would be associated with the physicochemical properties of propylene glycol and/or its poor ability to disassemble the conformational structure of amylose.

Based on the visual inspection and rheological measurements, seven samples were chosen for aerogel production, which are assigned with codes and summarized in Table 2.

### 2.4. Textural Properties of Aerogels

The influence of starch concentration and solvent-to-DMSO mass ratio on the textural properties of aerogels was investigated, and the results are presented in Table 3. For the aerogels prepared with water, the envelope density increases with starch concentration, as expected. However, the volumetric shrinkage shown no correlation with the envelope density, and the values were statically similar regardless of processing conditions. Specific surface area varied from 78 to 144 m^2^ g^−1^, but no clear influence of starch concentration and solvent-to-DMSO mass ratio is observed. On average, we can conclude that solvent-induced gelation with water yielded aerogels with a specific surface area of 113 ± 20 m^2^ g^−1^ (*n* = 6). The results for the sample S_15__PG_50_ gelled with propylene glycol also fall within this range.

**Figure 6:**
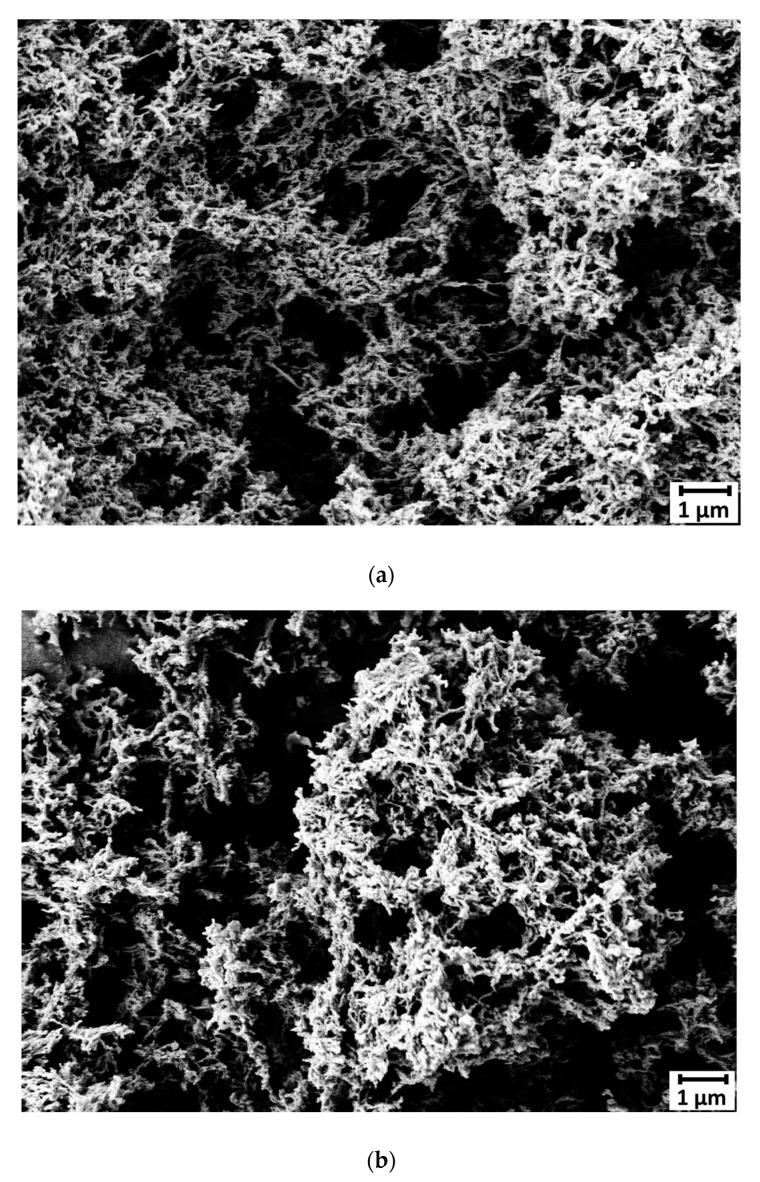
Scanning Electron Microscopy (SEM) images of samples (**a**) S_7.5__WA_70_ and (**b**) S_7.5__WA_80_.

Figure 6 shows SEM images from aerogels samples S_7.5__W_70_ and S_7.5__W_80_. Visual inspection of images reveal that the aerogels present a net-like structure with nonuniform pore sizes and no sign of remaining granules. The structure is essentially similar to starch aerogels prepared by pasting at 140 °C followed by a retrogradation step at 6 °C [3]. The specific surface area of the aerogels reported here is comparable with the values (60–100 m^2^ g^−1^) reported by other authors [8,9,15,31,32]. Higher values (183–254 m^2^ g^−1^) have been reported by [3,12,33], which are all produced by traditional pasting in water above 100 °C.

## 3. Conclusions

In this work, phase transitions from liquid to gel, in starch/DMSO mixtures, were systematically investigated as a function of starch concentration, type of solvent, and solvent-to-DMSO mass ratio. Based on the HSP and rheological measurements, it was demonstrated that hydrogen bonding and starch concentration play a crucial role on gel formation. We demonstrated that starch gels could be obtained from the solvent-induced gelation route, including a water-free system, and converted into aerogels. The resulting aerogels possessed comparably high surface areas and a well-connected fibrillar network, indicating that DMSO efficiently promoted the starch granules disruption. Further work evaluating other solvents with high values of total solubility parameters (*δ_t_*) or hydrogen bond interactions (*δ_h_*) will be carried out with the aim to find a gelation sphere for starch in the 3D Hansen space. This will provide information for predicting starch gelation in solvent systems that are miscible in sc-CO_2_ and would allow the direct drying of the gel. In addition, different starch types and amylose/amylopectin ratios could have an influence on the gelation profile of starch and will be considered in our future work with a long-term-goal to extend the list of biopolymers suitable for the water-free solvent-induced gelation.

## 4. Materials and Methods

### 4.1. Reagents

Corn starch (Hylon VII^®^, amylose content of 70%, density: 1.5095 g cm^−3^) was kindly provided by Ingredion (São Paulo, Brazil). DMSO and anhydrous ethanol (99.8% purity, denatured with 1% methyl ethyl ketone) were purchased from Carl Roth GmbH (Karlsruhe, Germany). Propylene glycol was provided by Sigma Aldrich (Steinheim, Germany). Glycerol was purchased from Merck (Hohenbrunn, Germany). Sulfolane was obtained from Alfa Aesar (Karlsruhe, Germany) and 2-dimethyl ethanolamine was obtained from Fluka Analytical (Munich, Germany). Deionized water was used in all experiments. Carbon dioxide (CO_2_) was supplied by AGA Gas GmbH (Hamburg, Germany).

### 4.2. Sample Preparation

To investigate the gelation behavior of starch, several samples were prepared with different starch concentrations (3, 5, 7.5, 10 and 15 wt%) and solvent-to-DMSO mass ratios (20:80, 30:70, 50:50, 70:30, and 80:20 *w*/*w*). The solvents used in this study were selected by their capacity to form hydrogen bonds and included water, glycerol, propylene glycol, sulfolane, and 2-dimethyl ethanolamine.

Before the experiments, a stock solution of starch solubilized in DMSO was prepared according to the methodology of Han and Lim (2004) [24] with some modifications. Briefly, starch was dispersed in DMSO (up to 30–50 wt% starch content). The mixture was heated at 80 °C under magnetic stirring until complete dissolution of granules (approximately 30 min), which was monitored by optical microscopy (VWR^®^ Compound Laboratory Microscopes, Leuven, Belgium). Then, the stock solution was stored at room temperature for 24 h to stabilize before use.

The samples were prepared as follows: the stock solution was mixed with pure DMSO and the solvent in the respective amounts to achieve a target starch concentration and a target solvent-to-DMSO mass ratio. The mixture was homogenized for 5 min at room temperature using magnetic stirring. After that, the solution was poured into glass tubes and stored at room temperature during 24 h for gel hardening.

### 4.3. Qualitative Assessment of Samples

After the hardening period, the glass tubes were tilted and the flow behavior of the samples were evaluated and classified as liquid (fluidly flowed sample), thick liquid (slowly flowed sample), or gel-like (not flowed sample). Gel-like samples were further studied: they were again prepared as described in Section 4.2, and the obtained solution was poured into cylindrical molds (diameter = 1.20 cm, length = 2.5 cm) instead of vials to form monoliths. After the hardening period of 24 h, the monoliths were removed from the molds and placed on a glass surface. Samples that were able to preserve its monolithic form were classified as strong gels.

### 4.4. Rheological Measurements

The gelation behavior of starch was investigated by oscillatory measurements using a rheometer Kinexus pro+ (Malvern Instruments, Worcestershire, UK). Isothermal analyses were conducted for 2 h at 20 °C with a fixed frequency of 1 Hz and a constant deformation of 0.5% using a cone-plate geometry of 20 mm diameter and 0.5° angle. The gap was adjusted to 0.8 mm. Tests were performed at least in duplicate.

### 4.5. Calculation of Hansen Solubility Parameters (HSP) for the Solvent/DMSO Mixtures

The total solubility parameter of the mixture (*δ_t,m_*) was calculated according to Equation (1), where *V* is the volume fraction (see calculations of volume fractions in the Supplementary Materials) and *δ_t_* is the total solubility parameter of the solvent:

(1) $$\delta_{\;t,m}=\sum^{\;}V_{n}\delta_{t,n}$$

The individual solubility parameters of the mixtures (*δ_d,m_*, *δ_p,m_*, *δ_h,m_*) were calculated similarly, according to Equations (2)–(4):

(2) $$\delta_{\;d,m}=\sum^{\;}V_{n}\delta_{d,n}$$

(3) $$\delta_{\;p,m}=\sum^{\;}V_{n}\delta_{p,n}$$

(4) $$\delta_{\;h,m}=\sum^{\;}V_{n}\delta_{h,n}$$

The HSP parameters of each solvent were obtained from [29] and are shown in Table 1.

### 4.6. Preparation of Aerogel Monoliths

Aerogels in the form of monoliths were prepared as follows: gel monoliths (Section 4.2 and Section 4.3) were submitted to solvent exchange with ethanol to remove the gelling solvents from the gel pores, due to their low solubility in supercritical CO_2_. For that, monoliths were soaked twice in anhydrous ethanol (gel-to-ethanol ratio 1:20 *v*/*v*) for 6 h (first step) and then 18 h (second step) at room temperature. The solvent exchange was completed when the solution density presented a value near to pure ethanol density (0.789 g cm^−3^), which was monitored by using a density meter (DMA 4500, Anton Paar, Austria) (Subrahmanyam et al., 2015) [34].

After the solvent exchange, the monoliths were dried with supercritical carbon dioxide (sc-CO_2_) using the equipment previously described by [35]. Briefly, monoliths were wrapped in filter papers and placed into a preheated (50 °C) high-pressure autoclave with a volume of 250 mL. An amount of 20 mL of ethanol was added in the autoclave to avoid solvent evaporation from the gels surface and its shrinkage before exposure to sc-CO_2_. The autoclave was kept at 50 °C using a heating jacket. Preheated CO_2_ (40–50 °C) was introduced to reach 12 MPa. The outlet micro-metering valve was adjusted to a continuous CO_2_ flow rate of 40 g min^−1^. Constant pressure of 12 MPa was maintained by feeding fresh carbon dioxide into the autoclave. The extraction was run for 3 h, followed by a depressurization at the same CO_2_ flow rate within 30–40 min [36].

### 4.7. Aerogel Characterization

Volumetric shrinkage of aerogel samples was calculated according to Equation (5), where *V_hydrogel_* and *V_aerogel_* are the volumes of the hydrogel and aerogel monoliths, respectively:

(5) $$VS_{t}=\frac{\left(V_{hydrogel}-V_{aerogel}\right)}{V_{hydrogel}}\times 100\%.$$

The envelope density of aerogel samples was determined by their weight and volume with accuracies of ±0.0001 g and 0.005 mm, respectively. The specific surface area was determined by Brunauer–Emmett–Teller (BET) method using the nitrogen adsorption/desorption technique (Nova 3000e, Quantachrome GmbH and Co. KG, Odelzhausen, Germany). Prior to measurements, samples were degassed at 40 °C under vacuum for at least 24 h. Internal morphology of the aerogels after sputtering with gold was analyzed by Scanning Electron Microscope (SEM) (Leo 1530, Zeiss, Jena, Germany) operated at 5 keV.

## Figures and Tables

**Table 1:** Hansen Solubility Parameters (HSP) of solvents obtained from [29].

Solvent	*δ_d_* (MPa^1/2^)	*δ_p_* (MPa^1/2^)	*δ_h_* (MPa^1/2^)	*δ_t_* (MPa^1/2^)
Dimethyl sulfoxide	18.4	16.4	10.2	26.7
Water	15.6	16	42.3	47.8
Propylene glycol	16.8	9.4	23.3	30.2
Glycerol	17.4	12.1	29.3	36.2
Sulfolane	20.3	18.2	10.9	29.3
2-Dimethyl ethanolamine	16.1	9.2	15.3	24.0

**Table 2:** Samples selected for aerogel production.

Sample Code	Starch Concentration (wt%)	Solvent	Solvent-to-DMSO Mass Ratio
S_7.5__WA_70_	7.5	water	70:30
S_7.5__WA_80_	7.5	water	80:20
S_10__WA_50_	10	water	50:50
S_10__WA_70_	10	water	70:30
S_10__WA_80_	10	water	80:20
S_15__WA_50_	15	water	50:50
S_15__PG_50_	15	propylene glycol	50:50

**Table 3:** Textural properties of aerogels samples prepared with water and propylene glycol as solvents.

Sample	*ρ_env_* (g cm^−^^3^)	VS (%)	S_a_ [BET] (m^2^ g^−^^1^)
S_7.5__WA_70_	0.097 ± 0.007	22 ± 3	133
S_7.5__WA_80_	0.099 ± 0.002	25 ± 1	144
S_10__WA_50_	0.125 ± 0.002	21 ± 2	102
S_10__WA_70_	0.138 ± 0.003	25 ± 1	103
S_10__WA_80_	0.127 ± 0.001	28 ± 5	92
S_15__WA_50_	0.200 ± 0.01	24 ± 1	107
S_15__PG_50_	0.195 ± 0.004	14 ± 1	78

*ρ_env_* = envelope density; VS = volumetric shrinkage, Equation (5); S_a_ = specific surface area.

## Supplementary Materials

The following are available online at https://www.mdpi.com/2310-2861/6/3/32/s1. Figure S1: G’ and G’’ profile of gel samples containing 5% of starch and different water-to-DMSO proportions: (a) 20:80, 30:70, and 50:50; (b) 70:30 and 80:20; Figure S2: G’ and G’’ profile of gel samples containing 7.5% of starch and different water-to-DMSO proportions: (a) 20:80, 30:70, and 50:50; (b) 70:30 and 80:20; Figure S3: G’ and G’’ profile of gel samples containing 15% of starch and different water-to-DMSO proportions: (a) 20:80, 30:70, and 50:50; (b) 70:30 and 80:20. Table S1: Visual appearance of the samples prepared with different starch concentrations, type of solvent, and solvent-to-DMSO mass ratios; Table S2: Calculated solubility parameters of mixture (*δ_m_*) for different solvent/DMSO mixtures and visual appearance of samples; Supporting information: Volume fraction calculation for the solvent/DMSO mixtures. References [34] is cited in the supplementary materials.

## References

[1] Bemiller J.N., Whistler R.L., Bemiller J.N., Whistler R.L. (2009). Starch—Chemistry and Technology.

[2] Vamadevan V., Bertoft E. (2015). Structure-function relationships of starch components. Starch/Stärke.

[3] Baudron V., Gurikov P., Smirnova I., Whitehouse S. (2019). Porous starch materials via supercritical and freeze-drying. Gels.

[4] Mikkonen K., Parikka K., Ghafar A., Tenkanen M. (2013). Prospects of polysaccharide aerogels as modern advanced food materials. Trends Food Sci. Technol..

[5] Tadini C.C., Villar M.A., Barbosa S.E., García M.A., Castillo L.A., López O.V. (2017). Chapter 2—Bio-Based Materials from Traditional and Nonconventional Native and Modified Starches. Starch-Based Materials in Food Packaging.

[6] Zhu F. (2019). Starch based aerogels: Production, properties and applications. Trends Food Sci. Technol..

[7] García-González C.A., Alnaief M., Smirnova I. (2011). Polysaccharide-based aerogels—Promising biodegradable carriers for drug delivery systems. Carbohydr. Polym..

[8] Mehling T., Smirnova I., Guenther U., Neubert R.H.H. (2009). Polysaccharide-based aerogels as drug carriers. J. Non. Cryst. Solids.

[9] De Marco I., Reverchon E. (2017). Starch aerogel loaded with poorly water-soluble vitamins through supercritical CO_2_ adsorption. Chem. Eng. Res. Des..

[10] García-González C.A., Jin M., Gerth J., Alvarez-Lorenzo C., Smirnova I. (2015). Polysaccharide-based aerogel microspheres for oral drug delivery. Carbohydr. Polym..

[11] Gurikov P., Smirnova I. (2018). Non-Conventional Methods for Gelation of Alginate. Gels.

[12] Druel L., Bardl R., Vorwerg W., Budtova T. (2017). Starch Aerogels: A Member of the Family of Thermal Superinsulating Materials. Biomacromolecules.

[13] Glenn G.M., Irving D.W. (1995). Starch-Based Microcellular Foams. Am. Assoc. Cereal Chem..

[14] Wang Y., Wu K., Xiao M., Ri B., Su Y., Jiang F. (2018). Thermal conductivity, structure and mechanical properties of konjac glucomannan/starch-based aerogel strengthened by wheat straw. Carbohydr. Polym..

[15] Goimil L., Braga M.E.M., Dias A.M.A., Gómez-amoza J.L., Concheiro A., Alvarez-lorenzo C., De Sousa H.C., García-González C.A. (2017). Supercritical processing of starch aerogels and aerogel-loaded poly (ε-caprolactone) scaffolds for sustained release of ketoprofen for bone. Biochem. Pharmacol..

[16] Martins M., Barros A.A., Quraishi S., Gurikov P., Raman S.P., Smirnova I., Duarte A.R.C., Reis R.L. (2015). Preparation of macroporous alginate-based aerogels for biomedical applications. J. Supercrit. Fluids.

[17] Starbird R., García-González C.A., Smirnova I., Krautschneider W.H., Bauhofer W. (2014). Synthesis of an organic conductive porous material using starch aerogels as template for chronic invasive electrodes. Mater. Sci. Eng. C.

[18] Anas M., Gönel A.G., Bozbag S.E., Erkey C. (2017). Thermodynamics of Adsorption of Carbon Dioxide on Various Aerogels. J. CO2 Util..

[19] Chen X., Guo L., Chen P., Xu Y., Hao H., Du X. (2017). Investigation of the high-amylose maize starch gelatinization behaviours in glycerol-water systems. J. Cereal Sci..

[20] Subrahmanyam R., Gurikov P., Meissner I., Smirnova I. (2016). Preparation of biopolymer aerogels using green solvents. J. Vis. Exp..

[21] McGrane S.J., Mainwaring D.E., Cornell H.J., Rix C.J. (2004). The Role of Hydrogen Bonding in Amylose Gelation. Starch/Stärke.

[22] Antoniou E., Buitrago C.F., Tsianou M., Alexandridis P. (2010). Solvent effects on polysaccharide conformation. Carbohydr. Polym..

[23] Cheetham N.W.H., Tao L. (1998). Amylose conformational transitions in binary DMSO/water mixtures. Carbohydr. Polym..

[24] Han J., Lim S. (2004). Structural changes of corn starches by heating and stirring in DMSO measured by SEC-MALLS-RI system. Carbohydr. Polym..

[25] Ptaszek A., Ptaszek P., Dziubiński M., Grzesik N.M., Liszka-Skoczylas M. (2017). The effect of structural properties on rheological behaviour of starches in binary dimethyl sulfoxide-water solutions. PLoS ONE.

[26] Bonnet J., Suissa G., Raynal M., Bouteiller L. (2015). Organogel formation rationalized by Hansen solubility parameters: Influence of gelator structure. Soft Matter.

[27] Gao J., Wu S., Rogers M.A. (2012). Harnessing Hansen solubility parameters to predict organogel formation. J. Mater. Chem..

[28] Raynal M., Bouteiller L. (2011). Organogel formation rationalized by Hansen solubility parameters. Chem. Commun..

[29] Hansen C. (2012). Hansen Solubility Parameters: A User’s Handbook.

[30] Feller J.-F. (2014). Different Strategies for Ecoplastics Development. Environmental Impact of Polymers.

[31] De Marco I., Baldino L., Cardea S., Reverchon E. (2015). Supercritical gel drying for the production of starch aerogels for delivery systems. Chem. Eng. Trans..

[32] Ubeyitogullari A., Ciftci O.N. (2016). Formation of nanoporous aerogels from wheat starch. Carbohydr. Polym..

[33] García-González C.A., Smirnova I. (2013). Use of supercritical fluid technology for the production of tailor-made aerogel particles for delivery systems. J. Supercrit. Fluids.

[34] Subrahmanyam R., Gurikov P., Dieringer P., Sun M., Smirnova I. (2015). On the Road to Biopolymer Aerogels—Dealing with the Solvent. Gels.

[35] Selmer I., Kleemann C., Kulozik U., Heinrich S., Smirnova I. (2015). Development of egg white protein aerogels as new matrix material for microencapsulation in food. J. Supercrit. Fluids.

[36] García-González C.A., Camino-Rey M.C., Alnaief M., Zetzl C., Smirnova I. (2012). Supercritical drying of aerogels using CO2: Effect of extraction time on the end material textural properties. J. Supercrit. Fluids.

