# Supplementary material for: Starch-Based Aerogels Obtained via Solvent-Induced Gelation
Source: Gels. 2020 Sep 19;6(3):32. doi: 10.3390/gels6030032 (PMC7559039; doi:10.3390/gels6030032)
Supplement: Supplementary file 1 [file gels-06-00032-s001.pdf]

gels

MDPI

Supplementary Materials

Starch-Based Aerogels Obtained via Solvent-Induced Gelation

Mirelle Dogenski [1,*], Pavel Gurikov [2,*], Victor Baudron [3], J. Vladimir de Oliveira [1], Irina Smirnova [3] and Sandra R. S. Ferreira [1,*]

[1] Department of Chemical and Food Engineering, Federal University of Santa Catarina, 88040-900, Florianópolis, Brazil; jose.vladimir@ufsc.br

[2] Laboratory for Development and Modelling of Novel Nanoporous Materials, Hamburg University of Technology, Eißendorfer Straße 38, 21073, Hamburg, Germany

[3] Institute of Thermal Separation Processes, Hamburg University of Technology, Eißendorfer Straße 38, 21073, Hamburg, Germany; victor.baudron@tuhh.de (V.B.); irina.smirnova@tuhh.de (I.S.)

* Correspondence: mirelledogenski@gmail.com (M.D.); pavel.gurikov@tuhh.de (P.G.); s.ferreira@ufsc.br (S.R.S.F.); Tel.: +55-48-3721-2537 (S.R.S.F); +49 40-428-784-275 (P.G.); +55-48 3721-4069 (M.D.)

Table S1. Visual appearance of the samples prepared with different starch concentrations, type of solvent, and solvent-to-DMSO mass ratios.

Solv.-to-DMSO Mass Ratio	Water	Propylene Glycol	Glycerol	Sulfolane	2-Dimethyl Ethanolamine
20:80	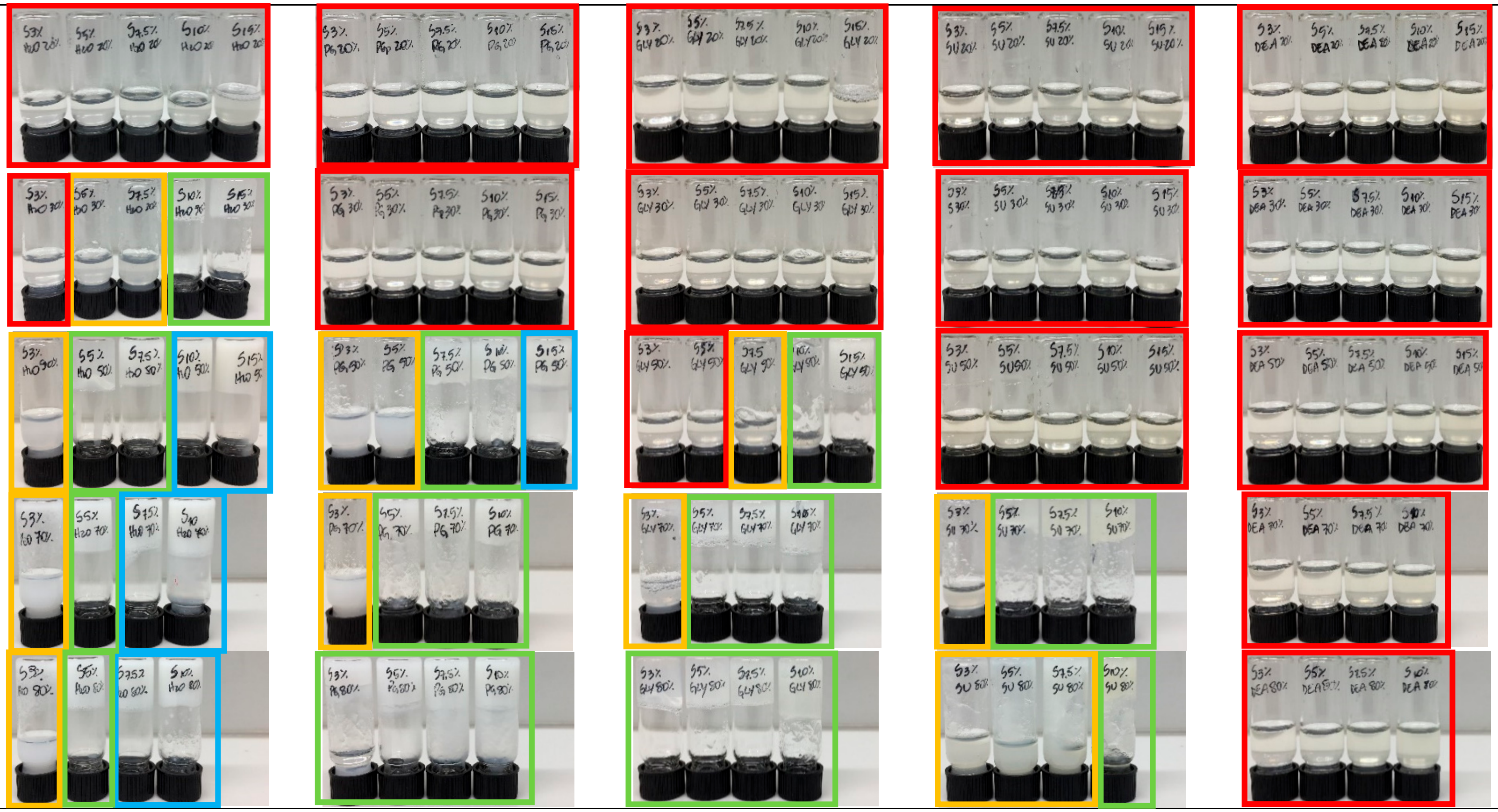				
30:70					
50:50					
70:30					
80:20					
Starch conc. (%)	3 5 7.5 10 15	3 5 7.5 10 15	3 5 7.5 10 15	3 5 7.5 10 15	3 5 7.5 10 15

Rectangles: red=liquid; yellow=thick liquid; green=gel-like; blue=strong gel

Volume fraction calculation for the solvent/DMSO mixtures

The volume fractions of the solvent/DMSO mixtures were obtained as follows:

For water as solvent: first, the density of water/DMSO mixtures (ρ) was calculated according to Equation (1) [34]:

$$c(\rho) = a_6 \times \rho^6 + a_5 \times \rho^5 + a_4 \times \rho^4 + a_3 \times \rho^3 + a_2 \times \rho^2 + a_1 \times \rho + a_0$$
$$for\ c < 82\%, \qquad (R^2 = 0{,}9990) \tag{1}$$

where a_0, a_1, a_2, a_3, a_4, a_5, and a_6 are the polynomial parameters and $c(\rho)$ is the weight concentration of DMSO. Then, the volume fractions were calculated according to Equation (2):

$$\rho = \rho_{DMSO} \times \frac{V_{DMSO}}{V} + \rho_{water} \times \frac{V_{water}}{V} \tag{2}$$

where ρ_{DMSO} and ρ_{water} are the densities of DMSO and water, respectively; $\frac{V_{DMSO}}{V}$ and $\frac{V_{water}}{V}$ correspond to the volume fractions of DMSO and water, respectively.

For the other solvents: the density of all other solvent/DMSO mixtures were calculated considering the volume additivity (Equation (3)), due to lack of experimental values or models in literature. Then, the volume fractions were obtained (Equation (4)):

$$V = \frac{m_{additive}}{\rho_{additive}} + \frac{m_{DMSO}}{\rho_{DMSO}} \tag{3}$$

$$v_i = \frac{V_i}{V} \tag{4}$$

where: $m_{solvent}$ and m_{DMSO}, are the mass of the solvent and DMSO, respectively; $\rho_{solvent}$ and ρ_{DMSO} are the density of the solvent and DMSO, respectively; V is the total volume of the mixture, V_i is the volume of compound i; v_i is the volume fraction of compound i.

Table S2. Calculated solubility parameters of mixture for different solvent/DMSO mixtures and visual appearance of samples.

Solvent	Sample	Solubility parameters of the mixture				Visual appearance				
	Solv.-to-DMSO Mass Ratio	$\delta_{d,m}$ (MPa$^{1/2}$)	$\delta_{p,m}$ (MPa$^{1/2}$)	$\delta_{h,m}$ (MPa$^{1/2}$)	$\delta_{t,m}$ (MPa$^{1/2}$)	Starch Concentration (wt.%)				
						3	5	7.5	10	15
Water	0:100	18.4	16.4	10.2	26.7					
	20:80	18.2	16.4	12.1	27.3	L	L	L	L	L
	30:70	18.1	16.4	14.1	28.1	L	TL	TL	GL	GL
	50:50	17.4	16.3	21.4	32.1	TL	GL	GL	SG	SG
	70:30	16.6	16.1	31.1	38.7	TL	GL	SG	SG	n.d.
	80:20	16.3	16.1	34.3	41.2	TL	GL	SG	SG	n.d.
	100:0	15.6	16.0	42.3	47.8					
Propylene glycol	0:100	18.4	16.4	10.2	26.7					
	20:80	18.1	14.9	12.9	26.8	L	L	L	L	L
	30:70	17.9	14.2	14.3	27.0	L	L	L	L	L
	50:50	17.6	12.8	16.9	27.6	TL	TL	GL	GL	SG
	70:30	17.3	11.4	19.5	28.5	TL	GL	GL	GL	n.d.
	80:20	17.1	10.7	20.8	29.0	GL	GL	GL	GL	n.d.
	100:0	16.8	9.4	23.3	30.2					
Glycerol	0:100	18.4	16.4	10.2	26.7					
	20:80	18.1	15.6	13.6	27.5	L	L	L	L	L
	30:70	18.0	15.2	15.4	28.1	L	L	L	L	L
	50:50	17.7	14.4	19.1	29.7	L	L	TL	GL	GL
	70:30	17.3	13.5	23.0	31.8	TL	GL	GL	GL	n.d.
	80:20	17.2	13.1	25.0	33.0	GL	GL	GL	GL	n.d.
	100:0	16.8	12.1	29.3	35.9					
Sulfolane	0:100	18.4	16.4	10.2	26.7					
	20:80	18.4	16.4	9.7	26.5	L	L	L	L	L
	30:70	18.4	16.5	9.4	26.4	L	L	L	L	L
	50:50	18.4	16.5	8.9	26.3	L	L	L	L	L
	70:30	18.4	16.5	8.3	26.1	TL	GL	GL	GL	L
	80:20	18.4	16.6	8.0	26.0	TL	TL	TL	GL	n.d.
	100:0	18.4	16.6	7.4	25.9					
2-dimethyl ethanolamine	0:100	18.4	16.4	10.2	26.7					
	20:80	17.9	14.7	11.4	25.8	L	L	L	L	L
	30:70	17.6	13.9	12.0	25.4	L	L	L	L	L
	50:50	17.1	12.4	13.0	24.8	L	L	L	L	L
	70:30	16.7	11.1	14.0	24.4	L	L	L	L	n.d.
	80:20	16.5	10.4	14.4	24.3	L	L	L	L	n.d.
	100:0	16.1	9.2	15.3	24.0					

L = liquid; TL = thick liquid; GL = gel-like; SG = strong gel; n.d = not determined.

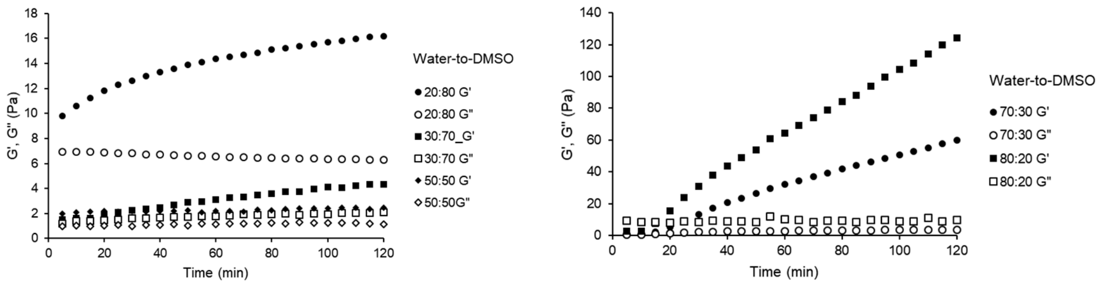

(**a**) (**b**)

Figure S1. G' and G'' profile of gel samples containing 5% of starch and different water-to-DMSO proportions: (**a**) 20:80, 30:70, and 50:50; (**b**) 70:30 and 80:20.

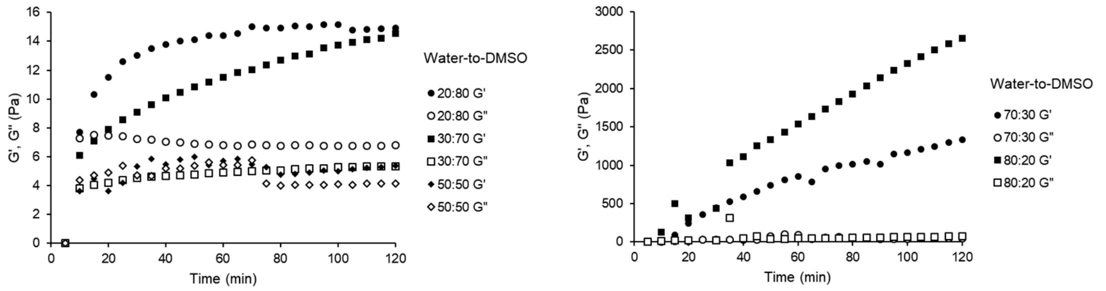

(**a**) (**b**)

Figure S2. G' and G'' profile of gel samples containing 7.5% of starch and different water-to-DMSO proportions: (**a**) 20:80, 30:70, and 50:50; (**b**) 70:30 and 80:20.

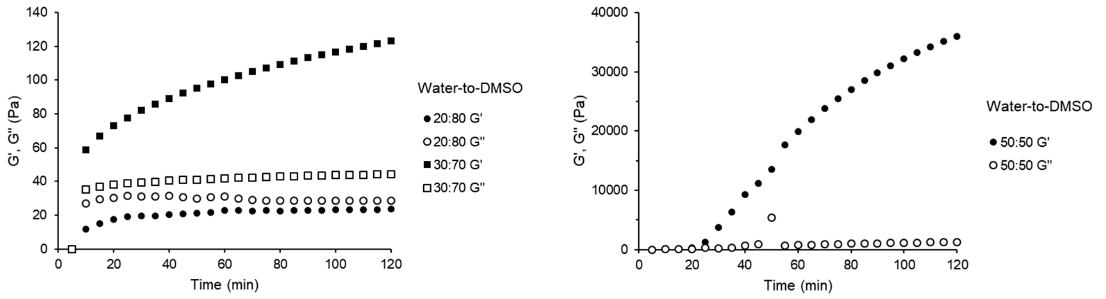

(**a**) (**b**)

Figure S3. G' and G'' profile of gel samples containing 15% of starch and different water-to-DMSO proportions: (**a**) 20:80, 30:70, and 50:50; (**b**) 70:30 and 80:20.

© 2020 by the authors. Licensee MDPI, Basel, Switzerland. This article is an open access article distributed under the terms and conditions of the Creative Commons Attribution (CC BY) license (http://creativecommons.org/licenses/by/4.0/).
